# Clinical Utility of Confirmatory Genetic Testing to Differentiate Sickle Cell Trait from Sickle-β^+^-Thalassemia by Newborn Screening

**DOI:** 10.3390/ijns6010007

**Published:** 2020-01-31

**Authors:** Lisa M. Shook, Deidra Haygood, Charles T. Quinn

**Affiliations:** 1 Ohio Department of Health Regional Sickle Cell Services Program–Region 1, Cincinnati, OH 45229, USA; 2 Cincinnati Comprehensive Sickle Cell Center, Cincinnati Children’s Hospital Medical Center, Cincinnati, OH 45229, USA; deidra.haygood@cchmc.org (D.H.); charles.quinn@cchmc.org (C.T.Q.); 3 Department of Pediatrics, University of Cincinnati College of Medicine, Cincinnati, OH 45267, USA; 4 Erythrocyte Diagnostic Laboratory, Cincinnati Children’s Hospital Medical Center, Cincinnati, OH 45229, USA

**Keywords:** genetic testing, sickle cell trait, sickle cell disease, sickle-β^+^-thalassemia, newborn screening for hemoglobinopathies

## Abstract

Hemoglobin separation techniques are the most commonly used laboratory methods in newborn screening and confirmatory testing programs for hemoglobinopathies. However, such protein-based testing cannot accurately detect several hemoglobinopathies in newborns, especially when β-thalassemia mutations are involved. Here, we describe a consecutive cohort of newborns who were identified by newborn screening to have a likely diagnosis of sickle-β^+^-thalassemia (having an “FSA” pattern) who were determined to have sickle cell traits by confirmatory and genetic testing. We illustrate the clinical utility of genetic testing to make a correct and timely diagnosis in the setting of newborn screening for hemoglobinopathies.

## 1. Introduction 

Hemoglobinopathies are a genetically complex group of blood disorders and the most commonly detected disorder overall by state newborn screening programs [1]. Since 2006, in the United States, all babies are tested for hemoglobinopathies as part of universal newborn screening [2]. Hemoglobinopathy diagnoses include sickle cell disease (SCD), thalassemia, compound heterozygous states, and many other hemoglobin (Hb) variants. Newborn screening follow-up protocols typically include confirmatory testing, parental education, and genetic counseling, particularly for babies with SCD. The benefits of universal newborn screening is that babies identified with SCD can be enrolled in a comprehensive SCD program and that penicillin prophylaxis can be initiated promptly to significantly reduce morbidity and mortality [3,4].

Protein-based testing for Hb disorders is used most commonly for both initial newborn screening and subsequent confirmatory testing. Protein-based testing includes different methods of Hb separation, such as electrophoresis (gel- or capillary-based), isoelectric focusing (IEF), and high-performance liquid chromatography (HPLC). However, a limitation of protein-based testing is that it cannot accurately detect many types of hemoglobinopathies in newborns, particularly when β-thalassemia mutations are involved.

A frequent newborn screening diagnostic scenario is the differentiation of the sickle cell trait (HbAS) from sickle β^+^-thalassemia (HbSβ^+^). While newborns with HbAS (sickle cell trait) have an “FAS” pattern on newborn screening, which indicates that Hb A is in higher abundance than Hb S, those with HbSβ^+^ typically have an “FSA” pattern, which indicates that Hb S is in higher abundance than Hb A. However, our recent clinical experience has been that an initial FSA pattern is much more likely to indicate a final diagnosis of HbAS than HbSβ^+^. This has direct implications for newborn screening follow-up, counseling and education, and the timeliness and accuracy of diagnosis and treatment. Here, we describe the problem we encountered and its solution by the addition of genetic testing to the confirmatory phase of newborn screening for hemoglobinopathies.

## 2. Methods

In the State of Ohio, universal newborn screening for hemoglobinopathies involves an initial test performed on dried blood spots collected at birth. This initial panel of testing is performed in a central laboratory by HPLC with confirmation by IEF. Hb patterns reported by the central laboratory correspond to absolute values of the abundance of Hb species and not proportional values or multiples of the median. Any newborn with a Hb variant (suspected disease, suspected trait, or unidentified variant) detected by this initial phase of testing then undergoes a second, confirmatory panel of testing performed using a new blood specimen obtained by venipuncture at an Ohio Department of Health (ODH) regional laboratory in the first 2–4 weeks of life. The laboratory for the ODH Regional Sickle Cell Services Program—Region 1 is housed within the Cincinnati Comprehensive Sickle Cell Center at Cincinnati Children’s Hospital Medical Center. Region 1 comprises 8 counties in southwestern Ohio in which approximately 500 babies have confirmatory testing for suspected Hb variants each year. Protein-based confirmatory testing using the second blood specimen includes capillary zone electrophoresis followed by, when appropriate, acid hemoglobin electrophoresis, IEF, or both. Genetic testing, when indicated, includes some combination of *HBA1, HBA2* and *HBB* sequence analysis and copy number variation analysis of the α-globin and β-globin gene clusters by multiplex ligation-dependent probe amplification. 

Here we studied a consecutive cohort of newborns with an “FSA” pattern (a suspected diagnosis of HbSβ^+^) on the initial newborn screening test who were born between July 2015 and August 2018 in Region 1 of the ODH Regional Sickle Cell Services Program. We compared the initial, suspected diagnosis of HbSβ^+^ (based on the initial, central testing using dried blood spots) to the diagnosis determined by confirmatory testing (using a second blood specimen) in the Region 1 laboratory using (1) protein-based Hb testing and (2) genetic testing, as described above. HbSβ^+^ was defined as the compound heterozygous state for the Hb S mutation and any β^+^-thalassemia mutation, whether substantiated by genetic testing, showing both *HBB* mutations and a normal *HBB* copy number; protein-based Hb testing, showing a markedly reduced abundance of Hb A and a higher abundance of Hb S than Hb A on a blood specimen obtained beyond the immediate neonatal period; or both genetic and protein-based methods.

During this study period, genetic testing was not universally applied in advance; it was used based on clinical suspicion and mostly after the protein-based Hb testing results were known. The results of this analysis were then used to change our standard clinical laboratory practice. This quality improvement project was exempt from formal institutional review board (IRB) review at Cincinnati Children’s Hospital Medical Center, and the requirement for written informed consent was waived.

## 3. Results

**Figure 1:**
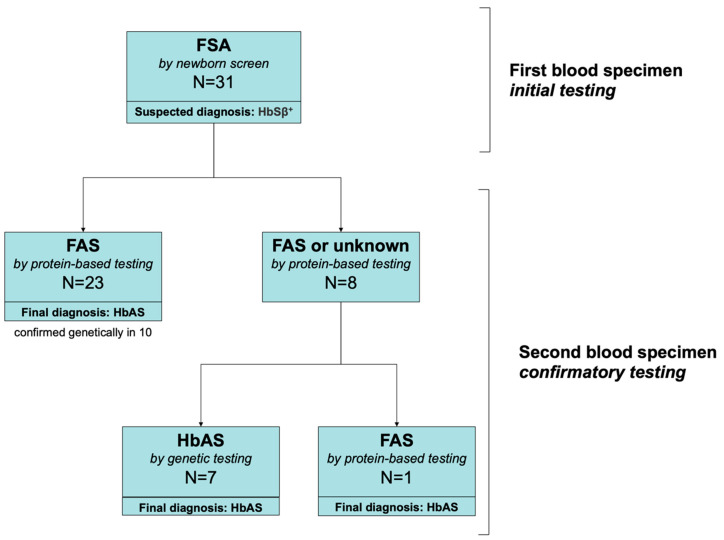
Suspected and final diagnosis by phase of testing.

Confirmatory testing using a second blood sample was performed for 1151 newborns with an abnormal Hb pattern between July 2015 and August 2018. Among these, we identified 31 who had a suspected diagnosis of HbSβ^+^ (an “FSA” pattern) on the initial newborn screening results from the central ODH laboratory. These 31 neonates were born at a mean estimated gestational age of 38.7 weeks (median 39; range 34–41). Only one was premature at 34 weeks. None was transfused before testing. Of these, 30 had protein-based confirmatory testing and 17 had genetic testing (Figure 1). On protein-based confirmatory testing at 2–4 weeks of age, 23/30 had an FAS pattern that indicated the correct diagnosis of HbAS (sickle cell trait), but 8/31 still had an FSA pattern consistent with HbSβ^+^. Of the 8 newborns who still had an FSA pattern, seven had genetic testing that established the correct diagnosis of HbAS; one newborn did not have genetic testing but had repeat protein-based testing at three months of age that established the correct diagnosis of HbAS. All newborns who had HbAS (*n* = 10) based on protein-based confirmatory testing who also had genetic testing had a final diagnosis of HbAS confirmed by genetic testing. During this study period, we identified one individual HbSβ^+^, but this newborn had an initial “FS” pattern (who, therefore, was not included in the “FSA” study population).

## 4. Discussion

In this consecutive cohort of 31 newborns with a suspected diagnosis of HbSβ^+^ based on initial newborn screening (an “FSA” pattern), none actually had HbSβ^+^. All had the sickle cell trait (HbAS), instead; that is, we found that an initial FSA pattern was much more likely to indicate a final diagnosis of HbAS than HbSβ^+^. Two-thirds of these newborns had a correct diagnosis of HbAS established at 2–4 weeks of age by protein-based confirmatory testing (and confirmed by genetic testing in a subset). However, the remaining one-third still had an incorrect, suspected diagnosis of HbSβ^+^ at 2–4 weeks of age. Without accurate and timely testing, this could lead to unnecessary treatment and testing of infants and incorrect, disease-focused counseling of parents and family members. Based on this experience in which genetic testing was not universally applied, we now perform simultaneous protein-based and genetic testing as our standard clinical practice in the panel of confirmatory tests (on the second blood specimen) for newborns who have an initial “FSA” pattern.

The accuracy of newborn screening for hemoglobinopathies can be improved by quantitative interpretation of Hb separation results using ratios or multiple of median cut-offs [5]. Alternatively, genetic testing can distinguish between HbAS and HbSβ^+^ (and identify any hemoglobinopathy) at any age, with a clinical laboratory turn-around time of 3–4 weeks (using our testing strategy). A benefit of genetic testing performed at the time of protein-based confirmatory testing for newborns with an FSA (or FS) patterns is that it can provide a timely, accurate diagnosis before there is a need to prescribe prophylactic antibiotics or be evaluated by a pediatric hematologist for a presumed, yet incorrect, diagnosis of SCD. This can improve the accuracy of newborn screening hemoglobinopathies, decrease the costs of newborn screening follow-up programs by preventing unnecessary clinic visits, and prevent unnecessary anxiety of parents and families by providing a correct diagnosis quickly.
